# Developing meaningful public involvement in a cancer clinical trials unit

**DOI:** 10.1186/1745-6215-16-S1-P10

**Published:** 2015-05-29

**Authors:** Jessica Baillie, Jim Fitzgibbon, Natalie Simon, Annmarie Nelson

**Affiliations:** 1 School of Healthcare Sciences, Cardiff University, Cardiff, CF14 4XN, UK; 2 Marie Curie Palliative Care Research Centre, School of Medicine, Cardiff University, Cardiff CF14 4YS, UK; 3 Involving People, National Institute for Social Care and Health Research Clinical Research Centre (NISCHR CRC), Cardiff, CF11 9LJ, UK

## Background

Public involvement in clinical trials has developed rapidly and is now expected by funders, research ethics committees, researchers and members of the public. Despite benefitting the research, researchers and public members involved, public involvement can be challenging and tokenistic. For several years, a cancer clinical trials unit (CTU) in Wales, in association with a government funded public involvement organisation, has made serious efforts to meaningfully and appropriately include members of the public across all its clinical trials.

## Method

With input from the aforementioned public involvement organisation, research infrastructure and cancer research institute, the CTU established an involvement group, overseen by a senior staff member. An experienced health research volunteer was appointed as Research Partner Coordinator to oversee and advocate for the public members (Research Partners), also acting as a contact for trial managers.

## Results

In total, 30 Research Partners have been recruited, with two individuals working on each clinical trial. The Research Partner Coordinator has developed terms of reference and standard operating procedures for recruitment, training and remuneration. Research Partners are offered comprehensive training through the public involvement organisation, and in-house training as required. Research Partners contribute to prioritising trials adopted by the CTU, study and protocol development, participant information sheets, publications, and Trial Management Group and Trial Steering Group meetings. Feedback is sought from Research Partners through questionnaires and optional debrief after each meeting, and periodic discussion sessions with the senior staff member and Research Partner Coordinator. An evaluation of CTU staff and Research Partner experience has been undertaken and will be reported separately.

## Conclusion

The CTU has sought to increase and improve inclusion of Research Partners across all its studies. Future planned CTU developments, which Research Partners will be involved with, include developing appropriate trial outcomes and ensuring patients’ understanding of clinical trials.

